# Dr. C. M. Fuller

**Published:** 1915-06

**Authors:** 


**Dr. C. M. Fuller,** (not listed in State Directory), died in Fairport, April 3, aged 86.

